# An Overview of RNA-Based Scaffolds for Osteogenesis

**DOI:** 10.3389/fmolb.2021.682581

**Published:** 2021-06-08

**Authors:** Laila A. Damiati, Sarah El-Messeiry

**Affiliations:** ^1^ Department of Biology, College of Science, University of Jeddah, Jeddah, Saudi Arabia; ^2^ Department of Genetics, Faculty of Agriculture, Alexandria University, Alexandria, Egypt

**Keywords:** bone osteogenesis, RNA, tissue engineering, gene therapy, CRISPR

## Abstract

Tissue engineering provides new hope for the combination of cells, scaffolds, and bifactors for bone osteogenesis. This is achieved by mimicking the bone’s natural behavior in recruiting the cell’s molecular machinery for our use. Many researchers have focused on developing an ideal scaffold with specific features, such as good cellular adhesion, cell proliferation, differentiation, host integration, and load bearing. Various types of coating materials (organic and non-organic) have been used to enhance bone osteogenesis. In the last few years, RNA-mediated gene therapy has captured attention as a new tool for bone regeneration. In this review, we discuss the use of RNA molecules in coating and delivery, including messenger RNA (mRNA), RNA interference (RNAi), and long non-coding RNA (lncRNA) on different types of scaffolds (such as polymers, ceramics, and metals) in osteogenesis research. In addition, the effect of using gene-editing tools—particularly CRISPR systems—to guide RNA scaffolds in bone regeneration is also discussed. Given existing knowledge about various RNAs coating/expression may help to understand the process of bone formation on the scaffolds during osseointegration.

## Introduction

Successful bone implants depend on well-established osteointegration which is highly relevant in implant design and/or coatings. Due to the bone complexity and dynamic structure, any large and unstable fractures may cause unsuccessful healing and require additional treatments before the bone regeneration occurs (Roseti et al., 2017). In tissue engineering, various scaffolding materials with different coatings have generated an enormous interest in developing an implants to match bone features (Leng et al., 2020). Implant characteristics, including the surface topography, chemistry, and mechanical properties, have a significant effect on osteogenesis and bacterial inhibition. For instance, nano-topographical surfaces, including nanorods, nanofibers, nanotubes, and nanowires, have demonstrated the ability to perform molecular-scale medical interventions for repairing damaged tissue (Bonilla-Represa et al., 2020). The possibility to functionalize the materials can be applied through different ways either physically such as surface wettability modification, or chemically, as with acid/alkaline treatment (Damiati et al., 2018).

Human bone mesenchymal stem cells (hBMSCs) are derived from the mesoderm during early embryonic development and are considered one of the most important seed cells for bone regeneration. The repair and regeneration of bone tissue is a complex procedure, and thus designing different biomaterials with load-related growth factors is one of the essential strategies in the bone regeneration field. For instance, bone morphogenic proteins (BMPs), including BMP2 (Damiati et al., 2018; Cheng et al., 2019), BMP3 (Daluiski et al., 2001), and BMP7 (Al-Jarsha et al., 2018), can induce stem cells differentiation into osteoblasts and chondrocytes. However, this approach also has some limitation in practical applications, such as the difficulty in transporting these growth factors to damaged areas and maintaining long-term high concentrations (Zhang et al., 2018). Due to that, the use of nucleic acids, including RNAs, as a bioactive coating for implants, has emerged recently and has been applied in bone implants (Miyamoto et al., 2018; Zhang et al., 2018).

RNA is a single strand molecule that forms secondary structures. RNA includes various types, such as messenger RNA (mRNA), which carries genetic information and form a protein as an end-product. Other non-protein codding RNA includes microRNA (miRNA), small interfering RNA (siRNA), and long noncoding RNA (lncRNA), which plays a more regulatory role in various cell functions (Mattick and Makunin, 2006).

In this review, we provide an overview of the effect of using different types of scaffolds based on RNAs family molecules as an organic coating, including mRNA, miRNA, siRNA, and lncRNA for bone formation applications. Further, the importance of using CRISPR based genome editing to guide the RNA for bone formation is also highlighted.

### Orthopedic Tissue Engineering

Tissue engineering is an emerging multidisciplinary science that combines molecular biology, engineering, and chemistry that aids in cellular *ex vivo* and *in vivo* tissue regeneration. Orthopedic tissue engineering in particular aims to fabricate new functional bone tissue by using combinations of cells and bioactive molecules (e.g., RNA coating) that are seeded onto biomaterials scaffolds to create an implantable “osteogenic” implant (Awad et al., 2014). However, these biomaterials can be used as implants in bone plates, dental implants, and joint replacement. Bone is considered the second most transplanted tissue after blood transfusion which increase the importance of finding the optimal biomaterial to be used clinically (Campana et al., 2014). Biomaterial scaffolds can generally be divided into natural (e.g., collagen and chitosan), synthetic (e.g., polymers), or metals (e.g., Ti, gold, and stainless steel), each with its own benefits and limitations.

These scaffolds should include few key elements to achieve regenerative bone, including bioactivity, which induces the formation of a direct chemical bond between the implant and host tissue; biocompatibility, which indicates an ability to perform with an appropriate host response in a specific application; and biodegradability, which indicates the ability to dissolve fully or partially when in contact with the living organism without causing any toxicity (Damiati et al., 2018). There are various material approaches that can be used to add bioactivity to bulk materials. Broadly, these are changes in the chemistry (Trino et al., 2018), stiffness (Behaviors et al., 2020) and topography (Hasan et al., 2017; Damiati et al., 2018; Behaviors et al., 2020). Different scaffolds have been utilized to facilitate the delivery of RNA, such as polymers-based scaffolds, ceramic-based scaffolds, and metal-based scaffolds. In the next sections, we will describe the pros and cons of these scaffolds in the bone regeneration field, then we will introduce the different types of RNAs as a novel organic coating material.

#### Polymer-Based Scaffolds

Polymers have been broadly used for fabricated medical devices and tissue-engineering scaffolds due to their unique properties such as high porosity, biodegradability, and their mechanical properties (Ji et al., 2006). There are two types of polymers, natural polymers and synthetic polymers. Natural polymers can be considered as the first biodegradable materials that were used in medical applications. They can be classified as: i. proteins, such as collagen, gelatin, keratin, actin, myosin, fibrinogen, and elastin; ii. polysaccharides, such as cellulose and chitin; and iii. polynucleotides such DNA and RNA (Dhandayuthapani et al., 2011; Chocholata et al., 2019). Natural polymers are commonly used due to their high biocompatibility and biodegradability as well as low antigenicity and inflammation. However, they have certain limitations, such as the low structural and mechanical properties, which requires combination with other materials for use in biomedical applications (Perez-Puyana et al., 2020).

Collagen is one of the natural scaffolds that has been extensively used for bone osteogenesis applications. Collage is a natural, biodegradable material that enhances cell attachment and migration, and does not cause any negative host immune responses. In bones, collagen is up to 89% of the organic matrix and 32% of the volumetric composition (O’Brien, 2011). However, collagen scaffolds have a poor compressive strength compared to native bone. Due to that, collagen is typically combined with another material to provide more structural rigidity (Ryan et al., 2019). Previous studies have shown that the compressive and tensile mechanical properties of collagen and glycosaminoglycan (a polysaccharide) can produce a highly porous collagen-GAG (CG) scaffold through physical and chemical cross-linking methods (Haugh et al., 2009; Tierney et al., 2009; Cunniffe and O’Brien, 2011). Additionally, another study by Ryan et al. showed that collagen scaffolds functionalized with copper-eluting glass were able to reduce the implant infections by *Staphylococcus aureus (S. aureus)* and to improve the osteogenesis and angiogenesis *in vitro* and *in vivo* (Ryan et al., 2019).

A combination between collagen and hydroxyapatite was able to activate the adipose-derived multipotent stromal cell (ASC) osteogenesis signaling pathway (Duan et al., 2017). In addition, in nature, cellulose is found as a mixture of crystalline and amorphous strictures that organized in a fringed fiber arrangement (Hearle, 1958). However, cellulose has been used in bone tissue engineering applications as the cellulous fibers to reassemble the collagen fibers of bone tissue. Shi et al. used the bacterial cellulose as delivery system to enhance the local concentration of cytokines, as the biocompatible scaffolds increased osteogenesis in the presence of BMP2 (Shi et al., 2012). Another study by Rescignano et al. used cellulose nanocrystals based on hydrogel composites and showed the ability to transport the biopolymeric nanoparticles to the bone marrow (Rescignano et al., 2014).

Synthetic polymers are very useful materials in biomedical applications due to their physical and mechanical properties that are similar to the natural polymers. In addition, synthetic polymers are much cheaper, and can be largely produced with a long-shelf time compared to the natural polymer’s scaffolds (Dhandayuthapani et al., 2011). However, synthetic polymers can be divided into two categories: degradable and non-degradable materials.

The biodegradable polymers illustrate the greatest applications in tissue engineering. Polylactide (PLA), polyglycolide (PGA), and poly(l-lactide-co-glycolide) (PLGA) are the most common synthetic polymers used in the tissue engineering due to their ability to adsorb water, hydrolysis, and the polymer chain enzymatic cleavage (Seal et al., 2001). Similar to the natural polymers, PLA has been tested with other materials to improve the mechanical properties, for instance PLA/hydroxyapatite (Holmes et al., 2016) and PLA/gelatin scaffolds (Ren et al., 2017). These combinations presented improvements in MSC cell adhesion and osteogenic differentiation. However, the most used synthetic polymer in bone regeneration applications is the PLGA (a linear copolymer that combines poly-l-lactic acid (PLLA) and PGA) due to the possibility of adjusting the degradation tunability rate. Again, and due to the poor mechanical properties, and low osteoconductivity, PLGA requires an additional material, like ceramics, or active glass to support the load-bearing application and cell differentiation (Pan and Ding, 2012; Gentile et al., 2014).

#### Ceramic-Based Scaffolds

Ceramic-based scaffolds are typically characterized by high mechanical stiffness (Young’s modulus) with very low elasticity and a hard, brittle surface. Hydroxyapatite (HA) and tricalcium phosphate (TCP) are communally used as ceramic scaffolds for bone regeneration applications. However, they showed excellent biocompatibility from bone applications viewpoint due to their chemical and structural similarity to the mineral of native bone. They have been used widely in dental and orthopedic surgery to fill bone defects and to coat a metallic implant to improve the cell-material interactions (O’Brien, 2011). However, they have certain limitations due to their difficulty of shaping, brittleness, and inability to control the degradation rate, and the new-formed bone between the HA porous material cannot sustain the required mechanical loading for bone remodeling (Wang, 2003).

#### Metal-Based Scaffolds

Metal-based scaffolds are extensively used as the best materials that provide stability and structural support which is essential for successful osseointegration. The commonly used biomaterials are titanium (Ti), Ti alloys, cobalt (co)-chromium (Cr) alloys, and stainless steel (Geetha et al., 2009). However, stiffness remains a significant limitation in the use of metals, as there is a huge gap between bone elasticity and the materials elasticity, which may lead to peri-implant resorption, implant loosing, and bone fracture (Shi L. et al., 2013). Another limitation of using metal biomaterials is the possibility to release some ions and/or particles through corrosion, which may lead to inflammation cascades and allergic reactions. Due to this, a proper treatment of the material surface is required to help to avoid these issues and to enhance the biocompatibility (Hallab et al., 2019).

Ti is considered the gold standard material in orthopaedical implants as it forms a very stable passive layer of TiO_2_ on its surface, which increases the osseointegration with bone. Due to this, Ti implants are used more commonly for the total joint replacements (Jäger et al., 2017).

Stainless steel is another commonly used material for implants; however, it has many drawbacks such as poor wear and corrosion resistance. Due to this, stainless steel is typically used for temporary implants or for long-term low-cost cemented implants. In addition, Co-Cr alloys showed an excellent wear, good corrosion resistance, and significant fatigue strength, which make these materials an ideal option for bearing surfaces (Navarro et al., 2008; Goriainov et al., 2014).

Generally, in bone tissue engineering and regenerative medicine research, there are three main approaches; i) cell therapy, where isolated cells are re-implanted in the defect sites to stimulate bone repair; ii) using a biomaterial scaffold, which help the endogenous cells to proliferate and differentiate, and iii) a combination of cells and biomaterial scaffolds which may also include using bioactive coatings (e.g., proteins like fibronectin and laminin, and growth factors, like BMP2 and BMP7) (Raftery et al., 2016; Damiati et al., 2018). However, the gene therapy and RNA interference (RNAi) has become the fourth approach to be involved the incorporation of regenerative stimuli into biomaterial scaffolds to enhance cell-material interactions (Raftery et al., 2016).

### Gene Therapy in Bone Repair

A promising advantages of gene therapy is the local delivery of gene sequence coding that has an ability to promote bone reparative processes. Recent studies have begun to provide potential evidence of gene therapies to deliver lasting therapeutic benefits for the bone and cartilage defects, with treatments focused mainly on the delivery of genes encoding for morphogenetic proteins (Evans and Huard, 2015). For instance, a direct injection of adenovirus carrying BMP2 presented significant repair of femoral defects in rodents (Betz et al., 2006).

Additionally, the direct delivery of recombinant adeno-associated viral vector (rAAV) with insulin-like growth factor 1 (IGF-1) (Cucchiarini and Madry, 2014), fibroblast growth factor 2 (FGF-2) (Cucchiarini et al., 2005), or SRY-related high mobility group-box gene9 (SOX9) (Cucchiarini et al., 2013), has shown an improvements in bone repair in rabbits. Various scaffolds have been used in gene combinations and gene recombinants through gene transfer using viral or non-viral vectors to target the relevant cells of osteochondral tissue engineering *in vivo* and *in vitro* (Madry et al., 2020). However, Table 1 summarizes some of the RNA-scaffolds matrix strength, weakness, opportunities, and threats (SWOT analysis) that should be taken into account before clinical use.

**TABLE 1:** The SWOT analysis of using scaffolds based on RNA-gene therapy.

Strengths	Weakness
• Easily to introduce into cells with high efficiency.	• Cells might not be transfectable.
• Can be rapidly produced in the laboratory.	• Non-renewable resource.
	• Virus-mediated toxic effects.
• Cost efficient.	• The uncertainty of the scaffold degradation rate may affect the efficacy of the RNAs.
• Chemical modification can be used to reduce the off-target effect.	
• May have a long-time effect.	• RNAs release limitation due to the strong interaction between scaffolds and the vectors.
• Scaffolds can protect RNA complexes from endogenous RNases.	
• The local RNA delivery into the site of interest may use to avoid unwanted release in other sites.	• Regulation policies may cause a delay to get clinical trials approvals.
**Opportunities**	**Threats**
• A new sector in the market to access that provides long-term revenue.	• Long-time follow-up is required to ensure the safety and efficacy of therapy.
• A collaboration between the digital market based on artificial intelligence (AI) and the currently available data may accelerate RNA treatment development.	• Pre- or post-immune reactivity may limit the clinical trials.
• Merge the field of personalized medicine and the gene therapy which targets the oligonucleotide of an individual’s genotype may become applicable for gene silencing and directing the gene-editing case.	• More studies are necessary to find the optimal RNA sequence to use for treatment.
	• Biosimilar competition will need to demonstrate the efficacy of new therapy comparing to the traditional therapies.
	• Significant investments are required to cover all the expenses needed for RNA-based therapy manufacturing.

### Different Types of RNAs in Mammalian Cells

Mammalian cells naturally contain a tremendous amount of various RNAs, which are involved in numerous complex tasks vital to the cells. The mRNA journey starts in the nucleus with DNA transcription followed by the processing of immature RNAs and ending with the export of mature RNAs to the cytoplasm to be translated into proteins (Lodish et al., 2000). RNAs that do not encode proteins but have functions are collectively known as non-coding RNAs (ncRNAs). There are two classes of ncRNAs housekeeping and regulatory ncRNAs. Housekeeping ncRNAs are expressed constitutively, including transfer ribonucleic acid (tRNA), ribosomal ribonucleic acid (rRNA), and small nuclear (snRNA). Many regulatory ncRNAs have been identified and have become a significant focus of research due to their role in gene regulation such as micro-RNA (miRNA), small interfering RNA (siRNA), small nucleolar RNA (snoRNA), Piwi-interacting RNA (piRNA), and long non-coding RNA (lncRNA) (Mattick and Makunin, 2006; Mercer et al., 2009; Ponting et al., 2009; Cech and Steitz, 2014).

**FIGURE 1:**
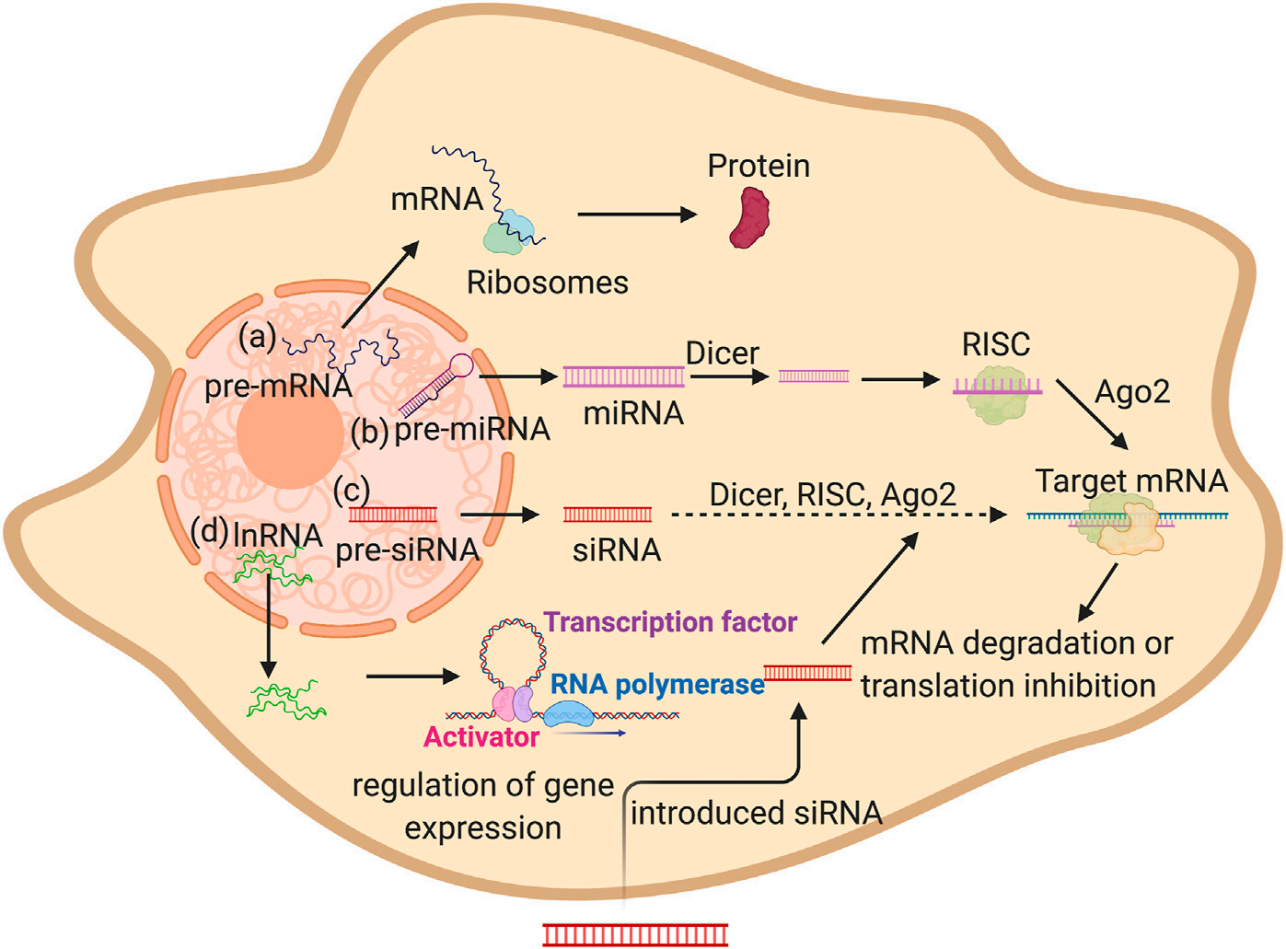
Schematic illustration showing different types of RNA in mammalian cells mRNA, miRNA, siRNA, and lnRN. **(A)** Premature mRNA gets exported to the cytoplasm then translated into protein by ribosomes. **(B,C)** pre miRNA and pre siRNA are produced in the nucleus and then gets exported to the cytoplasm then processed by Dicer followed by RISC complex formation, finally the miRNA or siRNA binds to the target sequence by complementation. This causes the degradation of the target RNA or translation block. **(D)** lncRNA are produced in the nucleus then exported into the cytoplasm in which they can regulate the gene expression (Created with BioRender.com).

The most common type of these RNA-delivered molecules is mRNA, which has been studied intensively. This RNA molecule is naturally synthesized in the nuclease as a pre-mRNA and is then processed and exported into the nucleus to be translated into proteins via the ribosome’s machinery (Figure 1A). Via the addition of a specific mRNA molecules into the cellular cytoplasm, certain proteins can be synthesized and supplemented for better bone osteogenesis, as seen by the addition of a chemically modified RNA encoding BMP2 gene to enhance bone regeneration (Elangovan et al., 2015).

Gene silencing pathways (RNA interference (RNAi)) is another type of mechanism in which short segments of RNA of around 22 nucleotides are introduced into the cells, similarly to siRNA, or produced naturally, as with certain siRNA and miRNA. These small nucleotide segments can alter the gene expression of a certain osteogenesis and bone differentiation related genes through the inhibition of gene expression. miRNAs are naturally synthesized in the nuclease as a single stranded RNA than can form a hairpin structure. They are exported into the cytoplasm and processed by DICER. They form the RNA-induced silencing complex (RISC) and then binds to the Ago2 protein. The targeted mRNA sequence by complete base paring finally inhibits the target gene expression through mRNA cleavage or the inhibition of protein translation (Figure 1B).

siRNA is a double stranded segment of RNA that works through partial binding of the mRNA targets, followed by mRNA cleavage via the RISC complex (Figure 1C) (Wilson and Doudna, 2013). RNAi has been widely introduced to cells as a therapeutic agent or for the inhibition of gene expression of a specific gene aiding in bone regeneration. lncRNAs are a group of RNAs transcribed in the nucleus with a length longer than 200 nucleotides. Some of these lncRNAs remains in the nucleus, while other are exported into the cytoplasm to play vital regulatory roles (Figure 1D). These RNA molecules play various roles, such as the regulation of gene expression and epigenetic regulation (Mercer et al., 2009). lncRNAs are also delivered into tissues to alter the gene expression of osteogenesis-related genes.

#### mRNA-Based Therapy

Advancements in the field of synthetic biology have enabled researchers to implement novel applications of artificial nucleic acid and its analogs as biomaterials. Synthesized mRNA can be delivered into cells for *in vitro* transcription (IVT mRNA) to repair and enhance bone regeneration using chemical or physical methods of delivery. They can be used to induce and modulate the expression of specific osteogenesis-related genes (Zhang et al., 2018; Leng et al., 2020). The host immune system can recognize the foreign mRNA, subsequently causing its degradation, and henceforth a chemical modification of its nucleic acids is required.

Elangovan and colleagues in 2015 successfully delivered the first chemically modified mRNA encoding BMP2 gene with a polyethylenimine polymer into BMSCs. They found a significant enhancement in bone regeneration *in vivo* with the chemically modified mRNA-polymer complex in a rat model with calvarial bone deficiency (Elangovan et al., 2015). Another study showed that the chemically modified mRNA encoding BMP2 and vascular endothelial growth factor (VEGF-A) genes in collagen-based scaffolds enhanced bone regeneration by driving bone osteogenesis in BMCs (Geng et al., 2021). Geng et al. found that a chemically modified mRNA encoding BMP9 in a collagen scaffold enhanced osteogenesis at a calvarial bone deficient site in rats (Geng et al., 2021). Serval other studies investigated the role of chemically modified mRNA BMP2 in osteogenesis *in vivo* and *in vitro*, showing that mRNA can be considered a very useful tool to enhance bone osteogenesis in the collagen or hydrogel-based scaffolds (Badieyan et al., 2016; Balmayor et al., 2017; Elangovan et al., 2015; Khorsand et al., 2017; Zhang et al., 2019).

#### RNAi-Based Therapy

Tissue engineering implements the organism’s own gene expression to aid in bone osteogenesis with the use of bone scaffolds. Therefore, using miRNA and siRNA can play vital roles in regulation of gene expression via the gene silencing pathway. They can be used as a biomolecule in bone tissue engineering by entering cells using a viral or a non-viral vectors such as lentivirus and Lipofectamine (Arriaga et al., 2019).

**FIGURE 2:**
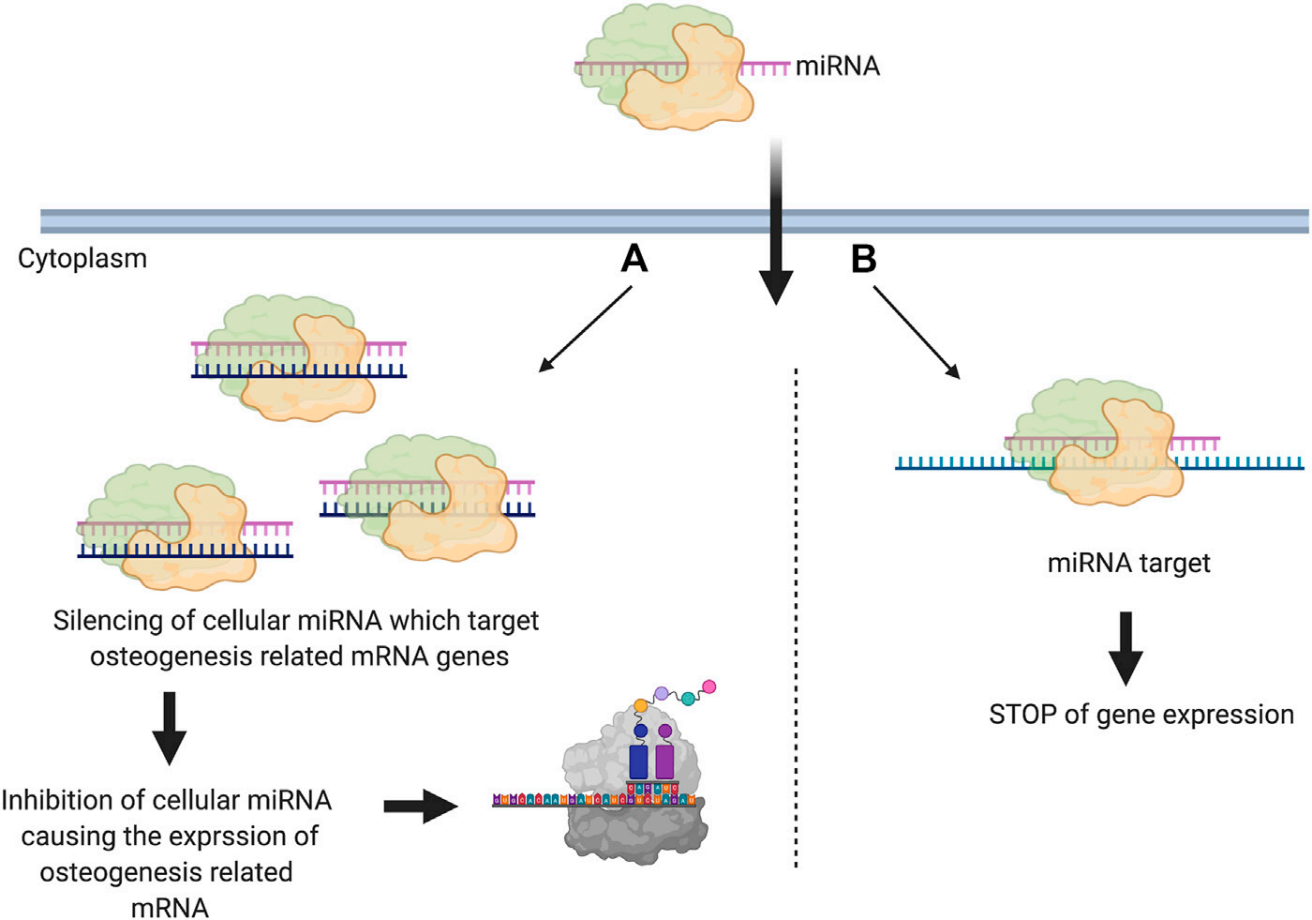
Schematic illustration showingthe two main used methods for miRNA mediated gene silencing in scaffold-based bone regeneration. miRNA is introduced into cells and works on silencing **(A)** cellular miRNA that can target osteogenesis related genes (anti-miRNA) **(B)** osteogenesis related genes (Created with BioRender.com).

miRNA-based therapy uses two main methods. The first is in silencing the cellular miRNA that binds to the target mRNA. In this method, the delivered miRNA binds by complementation to the cellular miRNA causing a loss of function. This subsequently causes the expression of the target gene (anti-miR) (Figure 2A). The second method is by direct down regulation of the gene via inducing the gene silencing pathway mediated by miRNA. In this case, the miRNA is designed to inhibit the target gene expression via complementary binding (Zhang et al., 2018) (Figure 2B).

Serval miRNAs (miR-100, miR-125b, miR-13, miR-196a, miR-218, and miR-22) were shown to promote osteogenesis through their action upon osteogenic target genes, while miR-126 was found to suppress osteogenesis. let-7b, let-7g, miR-133a and miR-29a were found to aid in collagen-fiber formation as summarized by (Sartori et al., 2019). miRNA can be added into the bone scaffolds in order to maintain stable long-lasting effects of these miRNAs upon the expression of the target RNA. The commonly used elements in miRNA scaffold-based tissue engineering are listed in Table 2, and Table 3 summarizing the effect of miRNA addition into scaffolds upon osteogenesis differentiation in MSCs (Leng et al., 2020).

**TABLE 2:** Summary of the main elements in miRNA-based scaffolds.

miRNA	Cells	Scaffolds
miR-26a	BMSCs, Adipose derived (ASCs), Bone marrow mononuclear cells (BMCs)	Poly glycerol sebacate
miR-135		Poly sebacoyl diglyceride
miR-148b		β -tricalcium phosphate
Anti-miR-31		Poly lactic-co-glycolic acid hydrogels
Anti-miR-34a		Hydroxyapatite containing scaffolds
Anti-miR-137		
Anti-miR-146a		
Anti-miR-221		
Anti-miR-335-5P		

**TABLE 3:** List of miRNA and role in osteogenesis differentiation in MSCs cells modulated by miRNA scaffold therapy.

Up- regulation	Target gene	Study	Down-regulation	Target gene	Study
miR-26a	Smad 1/5/8 (*drosophila* mothers against decapentaplegic)	Trompeter et al. (2013)	miR-26a	Osx through Gsk- β	Luzi et al. (2008)
				3 (glycogen synthase kinase) suppression	
miR-3960	BMP	Hu et al. (2011)	miR-93	Osx (osterix)	Yang et al. (2012)
miR-148B	NOG (noggin)	Mykhaylyk et al. (2008); Vosen et al. (2016)	miR-31		Baglìo et al. (2013)
miR-135	Smad 1/5/8	Vosen et al. (2016)	miR-214		Shi K. et al. (2013)
miR-31	Satb2 (special AT-rich sequence-binding protein 1)	Deng et al. (2013)	miR-637		Zhang et al. (2011)
miR-135	Hoxa2 (homeobox 2)	Xie et al. (2016)	miR-145		Jia et al. (2013)
miR-2861		Diomede et al. (2016)	miR-143		Li et al. (2014)
			miR-27a	Runx2 (runt-related transcription factor 2) through Hoxa 10 (homeobox a10) suppression	Godfrey et al. (2018)
			miR-23a	Runx2 through Satb2 suppression	Hassan et al. (2010)
			miR-27a		
			miR-24		
			miR-138	Runx2 through FAK (focal adhesion kinase) suppression	Qu et al. (2014)
			miR-34a	Runx2 through TAG1 (transient axonal glycoprotein 1) suppression	Chen et al. (2014)
			miR-22	Runx2 through HDAC6 (histone deacetylase 6) suppression	Huang et al. (2012)

Synthesized siRNA can also be used to silence specific osteogenesis-related genes. These double stranded siRNA could be introduced into the cells through lipid-based vectors, such as Lipofectamine. Other polymer-based delivery methods are available, such as the use of poly(lactic-co-glycolic) acid (PLGA), 3D polymeric hydrogels, and Atelocollagen scaffolds (Ghadakzadeh et al., 2016). These siRNA have shown to be a very useful tool in better understanding of osteogenesis genes, as seen in Table 4. The efficacy and lasting effects of the introduced siRNA were shown to increase in combination with scaffolds, such as lyophilized chitosan sponge (Ghadakzadeh et al., 2016).

**TABLE 4:** Examples of genes targeted by siRNA used to understand osteogenesis.

siRNA targeted gene	Finding	Study
S100A4	Silencing it induce osteogenic differentiation in periodontal ligament cells, via increase expression of osteoblastic markers (osteopontin and osteocalcin).	Kato et al. (2004)
Guanine nucleotide-binding protein (G protein) alpha subunit 1 (GNAS1)	Osteogenesis suppressor in MSCs, expression induction was detected by qRT-PCR and western blots of osteogenesis markers such as bone-specific sialoprotein (BSP), Cbfa1 and Osx.	Zhao and Ding (2007)
Nogging (NOG)	BMP2 expression increases causing induced osteoblastic differentiation in C2C12 cells, and enhance calvarial bone defects in rats.	Takayama et al. (2009); Nguyen et al. (2018)
NOG and GNAS	A high dose of BMP2, NOG, and GNAS delivery increased the cell death of human fetal osteoblast cell line (hFOB1.19) to more than 90% and the 50% less of cell proliferation comparing to the control.	Ramasubramanian et al. (2015)

The use of miRNA and siRNA in gene therapy has certain drawbacks: the small size of these RNA molecules leaves them unprotected from endogenous RNAase and prone to degradation; also, they also have an unstable structure and a short half-life. Therefore, chemical modifications are needed to protect them in the cells and to increase the stability, such as the use of a locked amino acid or the addition of 2-O-methoxyethyl phosphonothioate (2′-MOE) or cholesterol to modify the RNA (Zhang et al., 2018).

#### lncRNA-Based Therapy

lncRNA-based research has increased in the last few years as more functional roles of them have emerged. lncRNAs can either promote or inhibit the gene expression of serval genes or miRNAs (Ju et al., 2019). Studies have shown that lncRNA such as (MALAT1 (metastasis-associated lung adenocarcinoma transcript 1), HOTAIR (HOX transcript antisense RNA), H19, MODR, MIAT and MEG3) play essential roles in osteogenic differentiation. DANCE—another lncRNA—was found to regulate osteoclast differentiation in MSCs (Peng et al., 2018). Generally, lncRNAs are essential regulators for many biological processes; however, the exact roles of MSCs osteogenic differentiation remain unclear (Li et al., 2021).

The use of lncRNA combined with scaffolding has only been investigated in certain recent publications. Mingyue Wang et al. and Zheng et al. and revealed that the lncRNAs HIF1A-AS1 and PWRN1-209 promoted the bone formation of MSCs on Ti implants (Wang et al., 2020; Zheng et al., 2020). The lncRNA LOC103691336 was found to be upregulated in magnesium-based biodegradable implants, and competed with the BMP2 for miR-138-5p-binding in MSCs to change the inhibitory effect of miR-138-5p on BMP2 expression (Li et al., 2019).

In general, various RNAs molecules, such as mRNA, miRNA, siRNA, and lncRNA, can be implanted as biomolecules in different types of scaffolds to enhance the bone osteogenesis, and some examples are summarized in Table 5.

**TABLE 5:** RNA-based scaffolds used for bone osteogenesis.

Scaffolds	Cell type	Gene	Findings	Study
SMAT-Ti (surface mechanical attrition treatment)	hBMSCs	mRNA, miRNA, circRNA	The genes expression was upregulated (has-circ-0032599, has-circ-0032600, and has-circ-0032601) in SMAT-Ti scaffolds comparing to the annealed Ti.	Zhu et al. (2020)
Poly (ethylene glycol) (PEG)	hMSCs	miRNA, siRNA	Bone formation was improved in the rat calvarias bone defect after PEG gel implantation containing hMSCs and miRNA-20a compared to the hydrogels without siRNA or with negative control siRNA.	Nguyen et al. (2018)
3D hybrid scaffolds (Composite ink made of polycaprolactone (PCL)/ poly(D,L-lactide-co-glycolide) (PLGA)/ hydroxyapatite nano-particles	Rat bone marrow stem cells (rBMSCs)	miR-148b	*In vitro*: a significant upregulation of Runx2 levels for the miR-14b group comparing to the control, which indicates an early stage of bone differentiation during the bone remodeling, but not with osteocalcin (OCN) and alkaline phosphatase (ALP) expression.	Moncal et al. (2019)
			*In vivo*: the miR-148b supplemented scaffolds showed an effective modulation of rBMSCs and enhancing on the bone regeneration for the rat calvarial bone defects.	
ß-tricalcium phosphate (ß-TCP)	Mice bone marrow stem cells (mBMSCs)	miRNA-26a	The micro-computed tomography, eosin, and toluidine blue staining showed an improvement in the bone repair after ß-TCP scaffolds co-cultured with the MSCs. High expression for ALP, Runx2, and osteocalcin was also observed on the transfected implant.	Liu et al. (2018)
Chitosan (Cs)/ hyaluronic acid (HA) nanoparticles (NPS) cross linked onto gel culture plate	hBMSCs	miR-21	The combination of CS/HA/miR-21 NPs delivery on the hBMSCs sheets showed an improvement on the osteogenic differentiation markers (OCN and OPN) and enhanced the ALP activity, collagen secretion, and bone nodule formation.	Wang et al. (2016)
CS/nano HA/ nano-zirconium dioxide (nZrO_2_)	Mouse MSCs	miR-590-5p	The combination of CS/nHA/nZrO_2_/mBMSCs/ miR-590-5p suggested the potential of osteoconductive properties, by activating various signaling pathways, such as Runx2, Collagen type 1, and ALP.	Balagangadharan et al. (2018)
Collagen-nHA	hMSCs	miR-16	miR-16 may play an inhibitory role in osteogenesis due to its ability to directly target Smad5 and AcvR2a, which also could be used as a potential of a scaffold with the known potential for bone repair applications.	Mencia Castaño et al. (2019)
CS sponge	MSCs	siRNA	The CS sponge with siRNA significantly upregulated the OCN, ALP, and the vascular endothelia growth factor *in vitro*.	Jia et al. (2014)
			*In vivo*: the critical size defect in the rat skull showed a marked bone regeneration using the CS sponge and siRNA treatment.	
Collagen sponge	C2C12 cells (osteoblast)	siRNA	BMP2 enhanced the osteoblast differentiation by noggin-targeted siRNA *in vitro*.	Takayama et al. (2009)
			*In vivo*, the collagen-retaining BMP2 discs was implanted (after noggin-silencing siRNA) and the bone mineral contents were improved after 2 weeks of surgery.	
PEG/ poly (lactic acid)-dimethacrylate (PEG/PLA-GM) hydrogel	*In vivo* (mice)	siRNA	For the siRNA/NP that embedded within the gel, the diffusion could be controlled via encapsulation with tunable kinetics degradation and modeled for a delivery depot.	Wang et al., 2018)
Sand blasted, large-grit, acid-etched Ti (SLA-Ti)	hBMSCs	lncRNA	lncRNA PWRN1-209 enhanced ALP activity and osteogenic markers (e.g., Runx2, Col1, and Bsp) of MSCs cultured on microtopographic Ti comparing to the cells cultured on the flat Ti *in vitro.*	Wang et al. (2020)
SLA-Ti	hBMSCs	lncRNA	MSCs cultured on the SLA-TI scaffolds showed high levels of HIF1A-AS1 and VEGFA expression, while the knockdown of HIF1A-AS1 inhibited the osteogenic differentiation by regulating the p38 MPK cascade proteins.	Zheng et al. (2020)

### RNA Delivery

RNA delivery is a challenging task due to the following reasons; i: RNA molecules are negatively charged with a complex structure to pass across the cell membrane, and ii: the single stranded RNA is highly susceptible to degradation via endogenous cellular enzymes (Sahay et al., 2010). However, the use of RNA-based therapies has increased in the last few decades to repair bone defects. Due to the advancements in nanotechnology and molecular biology these RNA particles can be easily synthesized and delivered through various vectors into the targeted bone. The addition of these RNAs in the implant relays to the different indispensable roles in gene expression and regulation, including molecular triggers, signaling pathways, cellular processes, and the transcriptional regulators in bone osteogenesis (Zhang et al., 2018; Leng et al., 2020) (Table 6).

**TABLE 6:** State of significance, experimental challenges and prospects of mRNA-, RNAi-and lncRNA-based therapy for bone osteogenesis.

RNA family	mRNA	miRNA	siRNA	lncRNA
State of significance	mRNA has shown to be an extremely useful tool to enhance osteogenesis *in vivo* and *in vitro.*	Both can negatively and positively regulate osteogenesis and bone differentiation *in vivo* and *in vitro.*		Although most functions are still not fully understood, some lncRNAs play vital roles in regulation of osteogenesis.
Experimental challenges	Chemical modification of the mRNAs is needed and as they have short half-life (low stability).	miRNA complex affected genes pathways.	More investigations are required on siRNA sequences to confirm the current findings.	The roles of lnRNAs are still not fully understood.
Prospects	- Enhancement of delivery methods - Further investigations of other mRNA sequences encoding osteogenesis enhancement genes is needed.	- CRISPR/Cas9 technology can be used to silence siRNA genes or targeted genes will less off target effect in a more time efficient manner - CRISPR/Cas9 can also aid in better understanding of some miRNA and siRNA functions in osteogenesis by knock-down/off experiments.		Limited information is available. Advancement in RNA sequencing technology will reveal more functional roles in bone formation applications.

**FIGURE 3:**
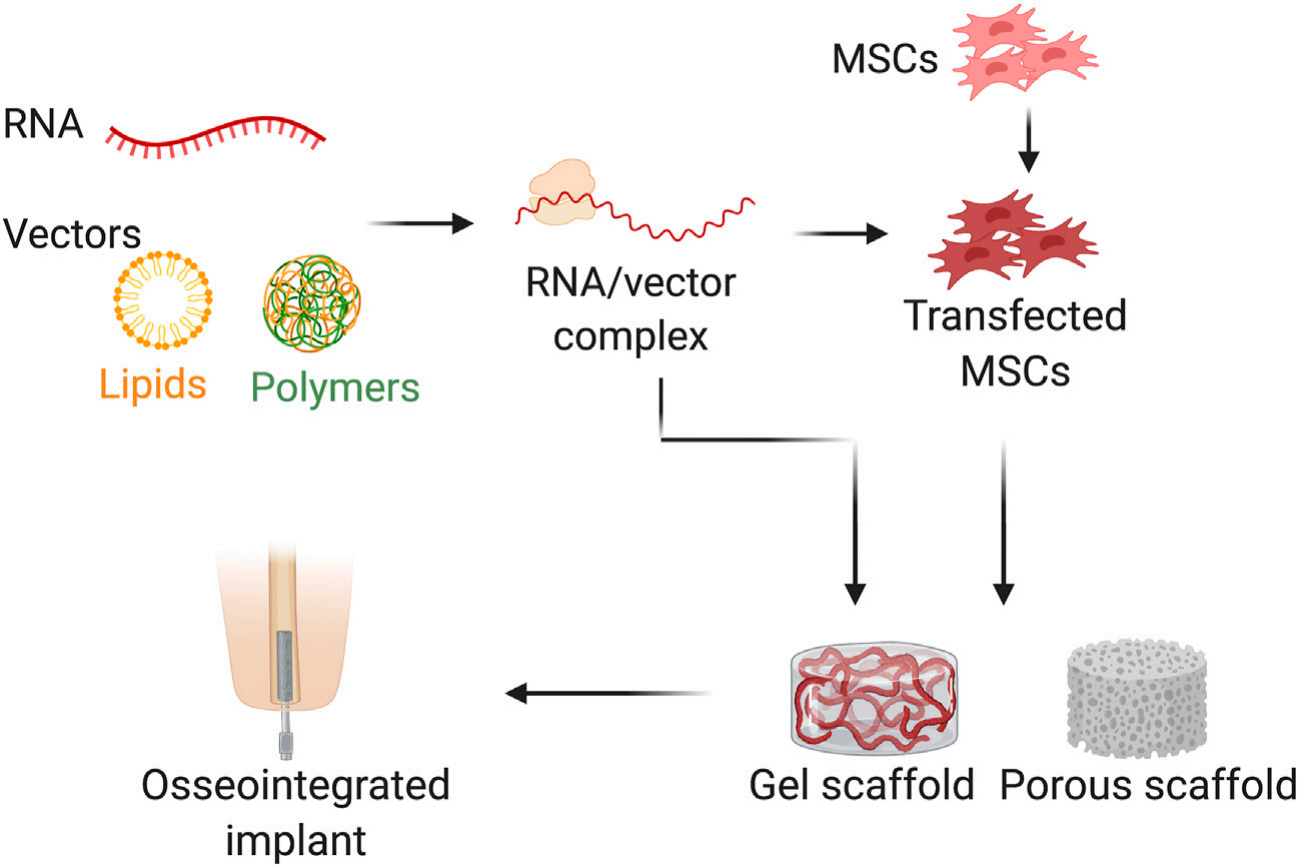
Schematic illustration of RNA delivery method used for bone osteointegration. RNA was combined with vector (e.g., lipids and polymers) and/or transfecting the MSCs before loaded to the scaffolds (e.g., gel or porous scaffolds) (Created with BioRender.com).

Two RNA delivery methods that are commonly used are systematic and local delivery. In systematic delivery, different vectors are used to deliver therapeutic RNA into scaffolds, such as viruses, dependent factors, or independent factors, like lipids, and polymers. In local delivery, the defect site primarily utilizes a non-viral biocompatible scaffold (Figure 3). For nanoparticles-specifically polymers-non-viral delivery is the most common method of RNA delivery due to the high ability to protect the RNA from degradation and to support the in-cellular uptake and endosomal escape (Anderson et al., 2003). Lipids and lipid-like materials are the second major approach of nanoparticle-based RNA delivery (Kaczmarek et al., 2017). Lipids are positively charged at acidic pH, which enhances the efficacy of endosomal escape (Schroeder et al., 2010), reducing the toxicity (Kanasty et al., 2013), and they have the capability to self-assemble into well-ordered nanoparticle structures called lipoplexes (Desigaux et al., 2007). In addition to the nanoparticles, for the direct conjugate a bioactive ligand such as N-acetylgalactosamine (Yu et al., 2016), antibodies (Xia et al., 2009), vitamins (Nishina et al., 2008), or cholesterol (Lorenz et al., 2004), can be used as an alternative method of RNA delivery. Additionally, another effective method of nucleic acid delivery are the chemical modifications made to the RNA itself that can impart degradation resistance to the RNAase, making them unrecognizable by the immune system (Soutschek et al., 2004; Morrissey et al., 2005). RNA chemical alterations to the ribose sugar, phosphate linkage, and individual bases can be used to deliver nucleic acids to the target receptors (Prakash et al., 2005; Wittrup and Lieberman, 2015; Li et al., 2016).

Several promising results have been found in various experimental studies implementing gene manipulated of MSC for treating bone defects, however these studies are still limited due to experimental caveats, and the safety and efficacy of the experiments need to be illustrated in the near future (Oryan et al., 2017). Also, developing a clinical-grade vector is a complicated, expensive process. No scaffold is currently in routine clinical use to deliver gene vector to the defect site. All the clinical trial results were not entirely satisfying, or were very limited to a few case studies, which require more investigations with longer follow-up (Kon et al., 2014; Madry et al., 2020).

### CRISPR to Guide RNA-Based Scaffolds

To obtain a successful bone implants in tissue engineering, all osteogenesis parameters are ought to be controlled and understood at molecular level. Traditional molecular methods can aid in this process; however, they have some limitations and require much experienced molecular biologist to obtain a genetically modified cell. MSCs are considered the primary used cell type used in studying bone regeneration and osteogenesis either to study the involved gene or to be included with scaffolds. However, some limitations were found in using it due to their ability to differentiate and the transplantation efficiency (Oryan et al., 2017; Arriaga et al., 2019). Henceforth, a novel and relatively easy genome editing approach has been implanted recently in the field of tissue engineering to control and understand osteogenesis at the molecular level. The bacteria adaptive immune system known as clustered regulatory interspaced short palindromic repeat (CRISPR) CRISPR-associated protein 9 (Cas9) (CRISPR/Cas9) has been mimicked recently to apply specific genome cuts in human cell lines (Yang et al., 2013).

**FIGURE 4:**
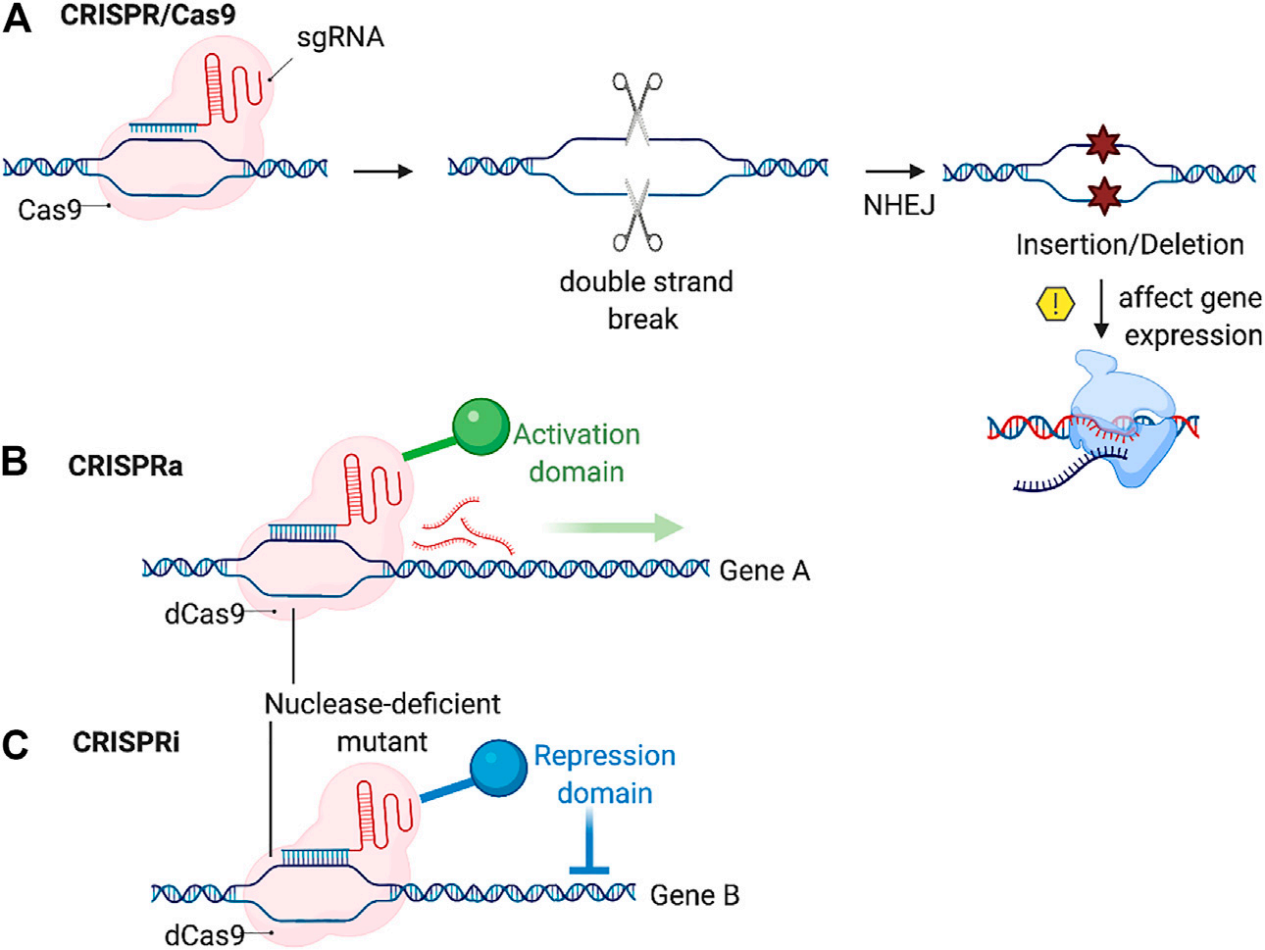
Schematic showing CRIPSR based genome editing. **(A)** CRISPR/Cas9 system works by the sgRNA recognize the target DNA then the Cas9 preform a double strand break, the NHEJ repair system then may causes insertion or deletion resulting in a change in the target gene expression. **(B,C)** CRISPRa/CRISPRi systems works by the sgRNA recognition of the target site followed by the activation or repression of gene expression of the target gene via an activation or a repression domain, altering the gene expression of the target gene (Created with BioRender.com).

This can occur by introducing into cells the Cas9 nuclease and a chimeric single guide RNA (sgRNA) complementary to the targeted genome segment, directed by the presence of the protospacer adjacent motif (PAM) sequence. The Cas9 nuclease guided by the sgRNA and the PAM sequence produces double strand breaks in the target genome sequence. The cells then repair this break via the non-homology end-joining pathway (NHEJ), which may result in a frame shift mutation (insertion/deletion) that can affect the gene expression of the targeted gene (Figure 4A). The high success rate, low-relative cost and low off-target effects made this system widely used by researchers to introduce specific cuts to the genome and to change the gene expression.

Several other types of gene editing methods have emerged adapting the CRISPR/Cas9 system, such as CRISPR interference (CRISPRi) and CRISPR activation (CRISPRa) relaying on the use of a modified Cas9 enzyme to alter the gene expression. CRISPRi works using a modified inactive Cas9 nuclease (dCas9) that blocks the targeted DNA transcription via sgRNA mediated binding. This results in silencing the targeted gene. On the other hand, CRISPRa stimulates gene expression of the target gene by fusion of the dCas9 with transcription activators, such as VP64, and this results in the gain of function of the targeted gene (Figures 4B,C) (Kampmann, 2018; Truong et al., 2019).

Other systems applied the CRISPR/Cas9 system for the live imaging of proteins, guided by the sgRNA to locate specific regions on the genome (Ma et al., 2018) as done by Narai et al. in which they used CRISPR technology to localize osteogenic differentiation in MSCs through the monitoring of bone gamma-carboxyglutamate protein (BGLAP) expression *in vivo* via an enhanced green fluorescent protein (EGFP) reporter (Narai et al., 2020).

#### CRISPR/Cas9 in Bone Osteogenesis

CRISPR/Cas9 gene silencing could be implemented to study the cellular control of osteogenesis genes, contributing to a better understanding of this vital cellular process.

A study by Lee el al. demonstrated that the CRISPR/Cas9-mediated gene silencing of PUMILIO2 (PUM2, a conserved posttranscriptional regulator) inhibited lipid accumulation and induced excessive bone formation by blocking MSC adipogenesis and enhancing the osteogenesis. They also showed that PUM2 works as a negative regulator on the 3′-untranslated regions of *janus kinase 2 (JAK2)* and *runt-relate transcription factor 2 (Runx2)* through direct binding (Lee et al., 2020).

The interaction between osteogenic and angiogenic cells has been considered for successful engineered vascularized bone tissue. However, based on Shahabipour et al.’s findings, the green fluorescent protein (GFP) knock-in umbilical vein endothelial cells (HUVECs) cell line via the CRISPR/Cas9 technology and cocultured with osteoblast-like cells (MG-63) within a 3D-fabricated hydrogel showed an improvement in the cell viability and angiogenic and osteogenic-related genes compared to the monocultured (Shahabipour et al., 2020).

In bone infections, CRISPR can be also implemented. As the implant/bone infections are a serious issue due to the antibiotic resistance, in particular for *Staphylococcus aureus (S. aureus)* and *Pseudomonas aeruginosa,* a new treatment has become essential for use in clinics (Damiati et al., 2018; Johnson et al., 2018). Cobb et al. used the CRISPR/Cas9 genomic editing tool to expand the temperate bacteriophage host range and enhance bactericidal activity through modification of the tail fiber protein. *In vitro*, they found the superiority of the phage to conventional vancomycin and fosfomycin antibiotics against *S. aureus* biofilms. However, *in vivo*, using the phage model with and without fosfomycin reduced the soft tissue infections but not the bone infections (Cobb et al., 2019).

#### CRISPRi and CRISPRa in Bone Osteogenesis

CRISPRi and CRISPRa have been used for different applications, such as genome-scale genetic screening (Bester et al., 2018), genetic interaction mapping (Du et al., 2017), cell signaling engineering (Liu et al., 2017), disease remodeling (Mandegar et al., 2016), and cell fate regulation (Black et al., 2016), also they can also be used to affect the gene expression of osteogenesis-related genes. Truong et al. developed a CRISPRai system that comprises active Cas9, activation/repression proteins complexes, and two single guide RNAs (sgRNAs) as a scaffold for recruiting activators (sgRNAa) or inhibitors (sgRNAi). They found that the CRISPR system delivered by the hybrid baculovirus stimulated chondrogenesis, and repressed the adipogenesis of rat BMSCs in 2D cultures, and stimulated the formation of engineered cartilage in 3D cultures, which may be of use to improve the calvarial bone healing (Truong et al., 2019). A more recent work by Hsu et al. showed that the hybrid baculovirus robustly activated endogenous *Wnt10b* and *Foxc2* for a long period of time and that the coactivation of *Wnt10b* and *Foxc2* successfully stimulated osteogenesis and repressed adipogenesis *in vitro*.

*In vivo*, the implantation of the CRISPRa-engineered BMSCs into the critical-sized calvarial defects in rat significantly improved bone healing (Hsu et al., 2020a). Another study from the same group reasoned that *Noggin gene* (*Nog)* inhibition, concurrent with BMP2 overexpression by using the CRISPRi system, could enhance the osteogenesis of adipose-derived stem cells and could improve calvarial bone healing (Hsu et al., 2020b).

There are some drawbacks to the use of CRISPR tools that can limit its *in vivo* applications, such as off-target effects if any of the sgRNAs were poorly designed. This could be avoided by the use of several sgRNAs for the same gene to increase the results validation or by using an enhanced version of Cas9 that has less off-target effects. Another tool is the use of a mutated Cas9 nuclease “Cas9 nickase (Cas9n)” that can induce a single strand break in two regions on the genome flanking the target gene sequence (Fu et al., 2013; Shen et al., 2014; Wu et al., 2019).

## Conclusion

Despite the rapid evolution in bone tissue engineering, many challenges need to be solved to find the optimal bone implants in clinical applications. Numerous materials have been utilized in bone tissue engineering applications such as polymers and metals, and each has benefits and limitations. However, Ti materials were demonstrated to be the best implants in orthopedical and dental applications *in vivo*, due to their biocompatibility and mechanical properties that are close to the human bones.

In recent decades, the RNA-based scaffolds have shown promising bone osteogenesis findings as therapeutic molecules coated or delivered to the scaffolds. In this review, we summarized the effects of different types of RNAs on the bone formation of different types of scaffolds. RNAs are starting to have a significant role as biomarkers for bone osteogenesis. A better understanding of RNA upregulation, downregulation, and silencing will increase bone remolding, improve treatments, and enhance patient quality of life by finding a better solution for implant loss.

We also discussed using the CRISPR-based genome editing technology, which offers a new tool to understand osteogenesis in many possible ways in a cost and time-efficient manner. CRISPR/Cas9 had proven to be a successful tool in understanding osteogenesis and bone healing, as well as providing a novel method to control bone infection. The utilization of this cutting-edge technology in the future will not only be limited to understand osteogenesis by obtaining a genetically modified cells (e.g., MSCs), but it will also provide a new tool in *in vivo* therapeutics gene editing in defective bone cells. Generally, this technology provides insights at the molecular and cellular level and aids in directing the cells cultured on the scaffolds to enhance bone formation, which provides a new technology to be used clinically for bone implants. Future applications based on RNA-scaffolds-cell interactions may accelerate bone osteogenesis and control implant failure.

## References

[B1] Al-Jarsha M. Moulisová V. Leal-Egaña A. Connell A. Naudi K. B. Ayoub A. (2018). Engineered Coatings for Titanium Implants to Present Ultra-Low Doses of BMP-7. ACS Biomater. Sci. Eng. 4 (5), 1812–1819. 10.1021/acsbiomaterials.7b01037 29862317 PMC5973637

[B2] Anderson D. G. Lynn D. M. Langer R. (2003). Semi-Automated Synthesis and Screening of a Large Library of Degradable Cationic Polymers for Gene Delivery. Angew. Chem. Int. Ed. 42 (27), 3153–3158. 10.1002/anie.200351244 12866105

[B3] Arriaga M. A. Ding M. H. Gutierrez A. S. Chew S. A. (2019). The Application of microRNAs in Biomaterial Scaffold‐Based Therapies for Bone Tissue Engineering. Biotechnol. J. 14 (10), e1900084. 10.1002/biot.201900084 31166084

[B4] Awad H. A. O’Keefe R. J. Lee C. H. Mao J. J. (2014). “Chapter 83 - Bone Tissue Engineering: Clinical Challenges and Emergent Advances in Orthopedic and Craniofacial Surgery,” in Principles of Tissue Engineering. 4th Edn, Editors (Lanza R. Langer R. Vacanati J. (Boston, MA: Academic Press), 1733–1743.

[B5] Badieyan Z. S. Berezhanskyy T. Utzinger M. Aneja M. K. Emrich D. Erben R. (2016). Transcript-Activated Collagen Matrix as Sustained mRNA Delivery System for Bone Regeneration. J. Control. Release 239, 137–148. 10.1016/j.jconrel.2016.08.037 27586186

[B6] Baglìo S. R. Devescovi V. Granchi D. Baldini N. (2013). MicroRNA Expression Profiling of Human Bone Marrow Mesenchymal Stem Cells during Osteogenic Differentiation Reveals Osterix Regulation by miR-31. Gene 527 (1), 321–331. 10.1016/j.gene.2013.06.021 23827457

[B7] Balagangadharan K. Viji Chandran S. Arumugam B. Saravanan S. Devanand Venkatasubbu G. Selvamurugan N. (2018). Chitosan/Nano-Hydroxyapatite/Nano-Zirconium Dioxide Scaffolds with miR-590-5p for Bone Regeneration. Int. J. Biol. Macromol. 111, 953–958. 10.1016/j.ijbiomac.2018.01.122 29415417

[B8] Balmayor E. R. Geiger J. P. Koch C. Aneja M. K. Van Griensven M. Rudolph C. (2017). Modified mRNA for BMP-2 in Combination with Biomaterials Serves as a Transcript-Activated Matrix for Effectively Inducing Osteogenic Pathways in Stem Cells. Stem Cell Dev. 26 (1), 25–34. 10.1089/scd.2016.0171 27676276

[B9] Behaviors B. Lan W. Huang T. Cho Y. Huang Y. (2020). Applied Sciences the Potential of a Nanostructured Titanium Oxide Layer with Self-Assembled Monolayers for Biomedical Applications : Surface Properties and Biomechanical Behaviors. Appl. Sci. 10, 590. 10.3390/app10020590

[B10] Bester A. C. Lee J. D. Chavez A. Lee Y.-R. Nachmani D. Vora S. (2018). An Integrated Genome-Wide CRISPRa Approach to Functionalize lncRNAs in Drug Resistance. Cell 173 (3), 649–664. 10.1016/j.cell.2018.03.052 29677511 PMC6061940

[B11] Betz O. B. Betz V. M. Nazarian A. Pilapil C. G. Vrahas M. S. Bouxsein M. L. (2006). Direct Percutaneous Gene Delivery to Enhance Healing of Segmental Bone Defects. J. Bone Joint Surg. Am. 88 (2), 355–365. 10.2106/00004623-200602000-00015 16452748

[B12] Black J. B. Adler A. F. Wang H.-G. D’Ippolito A. M. Hutchinson H. A. Reddy T. E. (2016). Targeted Epigenetic Remodeling of Endogenous Loci by CRISPR/Cas9-Based Transcriptional Activators Directly Converts Fibroblasts to Neuronal Cells. Cell Stem Cell 19 (3), 406–414. 10.1016/j.stem.2016.07.001 27524438 PMC5010447

[B13] Bonilla-Represa V. Abalos-Labruzzi C. Herrera-Martinez M. Guerrero-Pérez M. O. (2020). Nanomaterials in Dentistry: State of the Art and Future Challenges. Nanomaterials 10, 1770. 10.3390/nano10091770 PMC7557393 32906829

[B14] Campana V. Milano G. Pagano E. Barba M. Cicione C. Salonna G. (2014). Bone Substitutes in Orthopaedic Surgery: from Basic Science to Clinical Practice. J. Mater. Sci. Mater. Med. 25 (10), 2445–2461. 10.1007/s10856-014-5240-2 24865980 PMC4169585

[B15] Cech T. R. Steitz J. A. (2014). The Noncoding RNA Revolution-Trashing Old Rules to Forge New Ones. Cell 157 (1), 77–94. 10.1016/j.cell.2014.03.008 24679528

[B16] Chen L. HolmstrØm K. Qiu W. Ditzel N. Shi K. Hokland L. (2014). MicroRNA-34a Inhibits Osteoblast Differentiation and *In Vivo* Bone Formation of Human Stromal Stem Cells. Stem Cells 32 (4), 902–912. 10.1002/stem.1615 24307639

[B17] Cheng Z. A. Alba-Perez A. Gonzalez-Garcia C. Donnelly H. Llopis-Hernandez V. Jayawarna V. (2019). Nanoscale Coatings for Ultralow Dose BMP-2-Driven Regeneration of Critical-Sized Bone Defects. Adv. Sci. 6 (2), 1800361. 10.1002/advs.201800361 PMC6343071 30693176

[B18] Chocholata P. Kulda V. Babuska V. (2019). Fabrication of Scaffolds for Bone-Tissue Regeneration. Materials 12 (4), 568. 10.3390/ma12040568 PMC6416573 30769821

[B19] Cobb L. H. Park J. Swanson E. A. Beard M. C. McCabe E. M. Rourke A. S. (2019). CRISPR-Cas9 Modified Bacteriophage for Treatment of *Staphylococcus aureus* Induced Osteomyelitis and Soft Tissue Infection. PLoS One 14 (11), e0220421. 10.1371/journal.pone.0220421 31756187 PMC6874295

[B20] Cucchiarini M. Madry H. Ma C. Thurn T. Zurakowski D. Menger M. D. (2005). Improved Tissue Repair in Articular Cartilage Defects *In Vivo* by rAAV-Mediated Overexpression of Human Fibroblast Growth Factor 2. Mol. Ther. 12 (2), 229–238. 10.1016/j.ymthe.2005.03.012 16043094

[B21] Cucchiarini M. Madry H. (2014). Overexpression of Human IGF-I via Direct rAAV-Mediated Gene Transfer Improves the Early Repair of Articular Cartilage Defects *In Vivo* . Gene Ther. 21 (9), 811–819. 10.1038/gt.2014.58 24989812

[B22] Cucchiarini M. Orth P. Madry H. (2013). Direct rAAV SOX9 Administration for Durable Articular Cartilage Repair with Delayed Terminal Differentiation and Hypertrophy *In Vivo* . J. Mol. Med. 91 (5), 625–636. 10.1007/s00109-012-0978-9 23149825

[B23] Cunniffe G. M. O’Brien F. J. (2011). Collagen Scaffolds for Orthopedic Regenerative Medicine. J. Miner. Met. Mater. Soc. 63 (4), 66–73. 10.1007/s11837-011-0061-y

[B24] Daluiski A. Engstrand T. Bahamonde M. E. Gamer L. W. Agius E. Stevenson S. L. (2001). Bone Morphogenetic Protein-3 is a Negative Regulator of Bone Density. Nat. Genet. 27 (1), 84–88. 10.1038/83810 11138004

[B25] Damiati L. Eales M. G. Nobbs A. H. Su B. Tsimbouri P. M. Salmeron-Sanchez M. (2018). Impact of Surface Topography and Coating on Osteogenesis and Bacterial Attachment on Titanium Implants. J. Tissue Eng. 9, 204173141879069. 10.1177/2041731418790694 PMC6088466 30116518

[B26] Deng Y. Wu S. Zhou H. Bi X. Wang Y. Hu Y. (2013). Effects of a miR-31, Runx2, and Satb2 Regulatory Loop on the Osteogenic Differentiation of Bone Mesenchymal Stem Cells. Stem Cell Dev. 22 (16), 2278–2286. 10.1089/scd.2012.0686 23517179

[B27] Desigaux L. Sainlos M. Lambert O. Chevre R. Letrou-Bonneval E. Vigneron J.-P. (2007). Self-Assembled Lamellar Complexes of siRNA with Lipidic Aminoglycoside Derivatives Promote Efficient siRNA Delivery and Interference. Proc. Natl. Acad. Sci. U.S.A. 104 (42), 16534–16539. 10.1073/pnas.0707431104 17923669 PMC2034216

[B28] Dhandayuthapani B. Yoshida Y. Maekawa T. Kumar D. S. (2011). Polymeric Scaffolds in Tissue Engineering Application: A Review. Int. J. Polym. Sci. 2011, 1–19. 10.1155/2011/290602

[B29] Diomede F. Merciaro I. Martinotti S. Cavalcanti M. F. Caputi S. Mazzon E. (2016). miR-2861 is Involved in Osteogenic Commitment of Human Periodontal Ligament Stem Cells Grown onto 3D Scaffold. J. Biol. Regul. Homeost Agents 30 (4), 1009–1018. 28078846

[B30] Du D. Roguev A. Gordon D. E. Chen M. Chen S.-H. Shales M. (2017). Genetic Interaction Mapping in Mammalian Cells Using CRISPR Interference. Nat. Methods 14 (6), 577–580. 10.1038/nmeth.4286 28481362 PMC5584685

[B31] Duan W. Haque M. Kearney M. T. Lopez M. J. (2017). Collagen and Hydroxyapatite Scaffolds Activate Distinct Osteogenesis Signaling Pathways in Adult Adipose-Derived Multipotent Stromal Cells. Tissue Eng. Part C: Methods 23 (10), 592–603. 10.1089/ten.TEC.2017.0078 28877641 PMC5653142

[B32] Elangovan S. Khorsand B. Do A.-V. Hong L. Dewerth A. Kormann M. (2015). Chemically Modified RNA Activated Matrices Enhance Bone Regeneration. J. Control. Release 218, 22–28. 10.1016/j.jconrel.2015.09.050 26415855 PMC4631704

[B33] Evans C. H. Huard J. (2015). Gene Therapy Approaches to Regenerating the Musculoskeletal System. Nat. Rev. Rheumatol. 11 (4), 234–242. 10.1038/nrrheum.2015.28 25776949 PMC4510987

[B34] Fu Y. Foden J. A. Khayter C. Maeder M. L. Reyon D. Joung J. K. (2013). High-Frequency Off-Target Mutagenesis Induced by CRISPR-Cas Nucleases in Human Cells. Nat. Biotechnol. 31 (9), 822–826. 10.1038/nbt.2623 23792628 PMC3773023

[B35] Geetha M. Singh A. K. Asokamani R. Gogia A. K. (2009). Ti Based Biomaterials, the Ultimate Choice for Orthopaedic Implants - A Review. Prog. Mater. Sci. 54 (3), 397–425. 10.1016/j.pmatsci.2008.06.004

[B36] Geng Y. Duan H. Xu L. Witman N. Yan B. Yu Z. (2021). BMP-2 and VEGF-A modRNAs in Collagen Scaffold Synergistically Drive Bone Repair Through Osteogenic and Angiogenic Pathways. Commun. Biol. 4 (1), 1–14. 10.1038/s42003-020-01606-9 33469143 PMC7815925

[B37] Gentile P. Chiono V. Carmagnola I. Hatton P. (2014). An Overview of Poly(Lactic-Co-Glycolic) Acid (PLGA)-Based Biomaterials for Bone Tissue Engineering. Int. J. Mol. Sci. 15 (3), 3640–3659. 10.3390/ijms15033640 24590126 PMC3975359

[B38] Ghadakzadeh S. Mekhail M. Aoude A. Hamdy R. Tabrizian M. (2016). Small Players Ruling the Hard Game: SiRNA in Bone Regeneration. J. Bone Miner. Res. 31 (3), 475–487. 10.1002/jbmr.2816 26890411

[B39] Godfrey T. C. Wildman B. J. Beloti M. M. Kemper A. G. Ferraz E. P. Roy B. (2018). The microRNA-23a Cluster Regulates the Developmental HoxA Cluster Function during Osteoblast Differentiation. J. Biol. Chem. 293 (45), 17646–17660. 10.1074/jbc.RA118.003052 30242124 PMC6231122

[B40] Goriainov V. Cook R. M. Latham J. G. Dunlop D. Oreffo R. O. C. (2014). Bone and Metal: An Orthopaedic Perspective on Osseointegration of Metals. Acta Biomater. 10 (10), 4043–4057. 10.1016/j.actbio.2014.06.004 24932769

[B41] Hallab N. J. Samelko L. Caicedo M. (2019). Implant Material Bio-Compatibility, Sensitivity, and Allergic Reactions. Handbook of Spine Technology. Editor Cheng B. (New York, NY: Springer International Publishing), 1–23.

[B42] Hasan J. Jain S. Chatterjee K. (2017). Nanoscale Topography on Black Titanium Imparts Multi-Biofunctional Properties for Orthopedic Applications. Sci. Rep. 7, 41118. 10.1038/srep41118 28112235 PMC5253769

[B43] Hassan M. Q. Gordon J. A. R. Beloti M. M. Croce C. M. Wijnen A. J. v. Stein J. L. (2010). A Network Connecting Runx2, SATB2, and the miR-23a 27a 24-2 Cluster Regulates the Osteoblast Differentiation Program. Proc. Natl. Acad. Sci. U.S.A. 107 (46), 19879–19884. 10.1073/pnas.1007698107 20980664 PMC2993380

[B44] Haugh M. G. Jaasma M. J. O'Brien F. J. (2009). The Effect of Dehydrothermal Treatment on the Mechanical and Structural Properties of Collagen-GAG Scaffolds. J. Biomed. Mater. Res. 89A (2), 363–369. 10.1002/jbm.a.31955 18431763

[B45] Hearle J. W. S. (1958). A Fringed Fibril Theory of Structure in Crystalline Polymers. J. Polym. Sci. 28 (117), 432–435. 10.1002/pol.1958.1202811722

[B46] Holmes B. Bulusu K. Plesniak M. Zhang L. G. (2016). A Synergistic Approach to the Design, Fabrication and Evaluation of 3D Printed Micro and Nano Featured Scaffolds for Vascularized Bone Tissue Repair. Nanotechnology 27 (6), 064001. 10.1088/0957-4484/27/6/064001 26758780 PMC5055473

[B47] Hsu M.-N. Huang K.-L. Yu F.-J. Lai P.-L. Truong A. V. Lin M.-W. (2020a). Coactivation of Endogenous Wnt10b and Foxc2 by CRISPR Activation Enhances BMSC Osteogenesis and Promotes Calvarial Bone Regeneration. Mol. Ther. 28 (2), 441–451. 10.1016/j.ymthe.2019.11.029 31882321 PMC7001053

[B48] Hsu M.-N. Yu F.-J. Chang Y.-H. Huang K.-L. Pham N. N. Truong V. A. (2020b). CRISPR Interference-Mediated Noggin Knockdown Promotes BMP2-Induced Osteogenesis and Calvarial Bone Healing. Biomaterials 252, 120094. 10.1016/j.biomaterials.2020.120094 32422495

[B49] Hu R. Liu W. Li H. Yang L. Chen C. Xia Z.-Y. (2011). A Runx2/miR-3960/miR-2861 Regulatory Feedback Loop During Mouse Osteoblast Differentiation. J. Biol. Chem. 286 (14), 12328–12339. 10.1074/jbc.M110.176099 21324897 PMC3069436

[B50] Huang S. Wang S. Bian C. Yang Z. Zhou H. Zeng Y. (2012). Upregulation of miR-22 Promotes Osteogenic Differentiation and Inhibits Adipogenic Differentiation of Human Adipose Tissue-Derived Mesenchymal Stem Cells by Repressing HDAC6 Protein Expression. Stem Cell Dev. 21 (13), 2531–2540. 10.1089/scd.2012.0014 PMC3424982 22375943

[B51] Jäger M. Jennissen H. Dittrich F. Fischer A. Köhling H. (2017). Antimicrobial and Osseointegration Properties of Nanostructured Titanium Orthopaedic Implants. Materials 10 (11), 1302. 10.3390/ma10111302 PMC5706249 29137166

[B52] Ji Y. Ghosh K. Shu X. Li B. Sokolov J. Prestwich G. (2006). Electrospun Three-Dimensional Hyaluronic Acid Nanofibrous Scaffolds. Biomaterials 27, 3782–3792. 10.1016/j.biomaterials.2006.02.037 16556462

[B53] Jia J. Tian Q. Ling S. Liu Y. Yang S. Shao Z. (2013). miR-145 Suppresses Osteogenic Differentiation by Targeting Sp7. FEBS Lett. 587 (18), 3027–3031. 10.1016/j.febslet.2013.07.030 23886710

[B54] Jia S. Yang X. Song W. Wang L. Fang K. Hu Z. (2014). Incorporation of Osteogenic and Angiogenic Small Interfering RNAs into Chitosan Sponge for Bone Tissue Engineering. Int. J. Nanomedicine 9, 5307–5316. 10.2147/IJN.S70457 25429217 PMC4242407

[B55] Johnson C. T. Wroe J. A. Agarwal R. Martin K. E. Guldberg R. E. Donlan R. M. (2018). Hydrogel Delivery of Lysostaphin Eliminates Orthopedic Implant Infection by *Staphylococcus aureus* and Supports Fracture Healing. Proc. Natl. Acad. Sci. U.S.A. 115 (22), E4960–E4969. 10.1073/pnas.1801013115 29760099 PMC5984524

[B56] Ju C. Liu R. Zhang Y.-W. Zhang Y. Zhou R. Sun J. (2019). Mesenchymal Stem Cell-Associated lncRNA in Osteogenic Differentiation. Biomed. Pharmacother. 115, 108912. 10.1016/j.biopha.2019.108912 31048188

[B57] Kaczmarek J. C. Kowalski P. S. Anderson D. G. (2017). Advances in the Delivery of RNA Therapeutics: From Concept to Clinical Reality. Genome Med. 9 (1), 1–16. 10.1186/s13073-017-0450-0 28655327 PMC5485616

[B58] Kampmann M. (2018). CRISPRi and CRISPRa Screens in Mammalian Cells for Precision Biology and Medicine. ACS Chem. Biol. 13 (2), 406–416. 10.1021/acschembio.7b00657 29035510 PMC5886776

[B59] Kanasty R. Dorkin J. R. Vegas A. Anderson D. (2013). Delivery Materials for siRNA Therapeutics. Nat. Mater 12 (11), 967–977. 10.1038/nmat3765 24150415

[B60] Kato C. Kojima T. Komaki M. Mimori K. Duarte W. R. Takenaga K. (2004). S100A4 Inhibition by RNAi Up-Regulates Osteoblast Related Genes in Periodontal Ligament Cells. Biochem. Biophysical Res. Commun. 326 (1), 147–153. 10.1016/j.bbrc.2004.11.010 15567164

[B61] Khorsand B. Elangovan S. Hong L. Dewerth A. Kormann M. S. D. Salem A. K. (2017). A Comparative Study of the Bone Regenerative Effect of Chemically Modified RNA Encoding BMP-2 or BMP-9. AAPS J. 19 (2), 438–446. 10.1208/s12248-016-0034-8 28074350 PMC5712477

[B62] Kon E. Filardo G. Perdisa F. Venieri G. Marcacci M. (2014). Clinical Results of Multilayered Biomaterials for Osteochondral Regeneration. J. Exp. Orthop. 1 (1), 10. 10.1186/s40634-014-0010-0 26914755 PMC4648845

[B63] Lee M. H. Wu X. Zhu Y. (2020). RNA‐Binding Protein PUM2 Regulates Mesenchymal Stem Cell Fate via Repression of JAK2 and RUNX2 mRNAs. J. Cell Physiol. 235 (4), 3874–3885. 10.1002/jcp.29281 31595981 PMC6944769

[B64] Leng Q. Chen L. Lv Y. (2020). RNA-Based Scaffolds for Bone Regeneration: Application and Mechanisms of mRNA, miRNA and siRNA. Theranostics 10 (7), 3190–3205. 10.7150/thno.42640 32194862 PMC7053199

[B65] Li B. Luo X. Dong Y. (2016). Effects of Chemically Modified Messenger RNA on Protein Expression. Bioconjug. Chem. 27 (3), 849–853. 10.1021/acs.bioconjchem.6b00090 26906521

[B66] Li D. Yu K. Xiao T. Dai Y. Liu L. Li H. (2019). LOC103691336/miR‐138‐5p/BMPR2 axis Modulates Mg‐Mediated Osteogenic Differentiation in Rat Femoral Fracture Model and Rat Primary Bone Marrow Stromal Cells. J. Cell Physiol. 234 (11), 21316–21330. 10.1002/jcp.28736 31081160

[B67] Li E. Zhang J. Yuan T. Ma B. (2014). miR-143 Suppresses Osteogenic Differentiation by Targeting Osterix. Mol. Cell Biochem. 390 (1), 69–74. 10.1007/s11010-013-1957-3 24381059

[B68] Li R. Zhang W. Yan Z. Liu W. Fan J. Feng Y. (2021). Long Non-Coding RNA (LncRNA) HOTAIR Regulates BMP9-Induced Osteogenic Differentiation by Targeting the Proliferation of Mesenchymal Stem Cells (MSCs). Aging 13 (3), 4199–4214. 10.18632/aging.202384 33461171 PMC7906180

[B69] Liu Y. Han J. Chen Z. Wu H. Dong H. Nie G. (2017). Engineering Cell Signaling Using Tunable CRISPR-Cpf1-Based Transcription Factors. Nat. Commun. 8 (1), 2095. 10.1038/s41467-017-02265-x 29235474 PMC5727435

[B70] Liu Z. Chang H. Hou Y. Wang Y. Zhou Z. Wang M. (2018). Lentivirus-Mediated microRNA-26a Overexpression in Bone Mesenchymal Stem Cells Facilitates Bone Regeneration in Bone Defects of Calvaria in Mice. Mol. Med. Rep. 18, 5317–5326. 10.3892/mmr.2018.9596 30365148 PMC6236311

[B71] Lodish H. Berk A. Zipursky S. (2000). Molecular Cell Biology. 5th Edn. New York, NY: W. H. Freeman.

[B72] Lorenz C. Hadwiger P. John M. Vornlocher H.-P. Unverzagt C. (2004). Steroid and Lipid Conjugates of siRNAs to Enhance Cellular Uptake and Gene Silencing in Liver Cells. Bioorg. Med. Chem. Lett. 14 (19), 4975–4977. 10.1016/j.bmcl.2004.07.018 15341962

[B73] Luzi E. Marini F. Sala S. C. Tognarini I. Galli G. Brandi M. L. (2008). Osteogenic Differentiation of Human Adipose Tissue-Derived Stem Cells Is Modulated by the miR-26a Targeting of the SMAD1 Transcription Factor. J. Bone Miner. Res. 23 (2), 287–295. 10.1359/jbmr.071011 18197755

[B74] Ma H. Tu L.-C. Naseri A. Chung Y.-C. Grunwald D. Zhang S. (2018). CRISPR-Sirius: RNA Scaffolds for Signal Amplification in Genome Imaging. Nat. Methods 15 (11), 928–931. 10.1038/s41592-018-0174-0 30377374 PMC6252086

[B75] Madry H. Venkatesan J. K. Carballo-Pedrares N. Rey-Rico A. Cucchiarini M. (2020). Scaffold-Mediated Gene Delivery for Osteochondral Repair. Pharmaceutics 12, 930. 10.3390/pharmaceutics12100930 PMC7601511 33003607

[B76] Mali P. Yang L. Esvelt K. M. Aach J. Guell M. DiCarlo J. E. (2013). RNA-Guided Human Genome Engineering via Cas9. Science 339 (6121), 823–826. 10.1126/science.1232033.RNA-Guided 23287722 PMC3712628

[B77] Mandegar M. A. Huebsch N. Frolov E. B. Shin E. Truong A. Olvera M. P. (2016). CRISPR Interference Efficiently Induces Specific and Reversible Gene Silencing in Human iPSCs. Cell Stem Cell 18 (4), 541–553. 10.1016/j.stem.2016.01.022 26971820 PMC4830697

[B78] Mattick J. S. Makunin I. V. (2006). Non-Coding RNA. Hum. Mol. Genet. 15, R17–R29. 10.1093/hmg/ddl046 16651366

[B79] Mencía Castaño I. Curtin C. M. Duffy G. P. O'Brien F. J. (2019). Harnessing an Inhibitory Role of miR-16 in Osteogenesis by Human Mesenchymal Stem Cells for Advanced Scaffold-Based Bone Tissue Engineering. Tissue Eng. Part A 25 (1–2), 24–33. 10.1089/ten.TEA.2017.0460 29490603

[B80] Mercer T. R. Dinger M. E. Mattick J. S. (2009). Long Non-Coding RNAs: Insights into Functions. Nat. Rev. Genet. 10 (3), 155–159. 10.1038/nrg2521 19188922

[B81] Miyamoto N. Yamachika R. Sakurai T. Hayakawa T. Hosoya N. (2018). Bone Response to Titanium Implants Coated with Double- or Single-Stranded DNA. Biomed. Res. Int. 2018, 1–11. 10.1155/2018/9204391 PMC6020655 30009177

[B82] Moncal K. K. Aydin R. S. T. Abu-Laban M. Heo D. N. Rizk E. Tucker S. M. (2019). Collagen-Infilled 3D Printed Scaffolds Loaded with miR-148b-Transfected Bone Marrow Stem Cells Improve Calvarial Bone Regeneration in Rats. Mater. Sci. Eng. C Mater. Biol. Appl. 105, 110128. 10.1016/j.msec.2019.110128 31546389 PMC6761997

[B83] Morrissey D. V. Lockridge J. A. Shaw L. Blanchard K. Jensen K. Breen W. (2005). Potent and Persistent *In Vivo* Anti-HBV Activity of Chemically Modified siRNAs. Nat. Biotechnol. 23 (8), 1002–1007. 10.1038/nbt1122 16041363

[B84] Mykhaylyk O. Zelphati O. Rosenecker J. Plank C. (2008). siRNA Delivery by Magnetofection. Curr. Opin. Mol. Ther. 10 (5), 493–505. 18830925

[B85] Narai T. Watase R. Nakayama Y. Kodani I. Inoue T. Kokura K. (2020). Establishment of Human Immortalized Mesenchymal Stem Cells Lines for the Monitoring and Analysis of Osteogenic Differentiation in Living Cells. Heliyon 6 (10), e05398. 10.1016/j.heliyon.2020.e05398 33163667 PMC7610338

[B86] Navarro M. Michiardi A. Castaño O. Planell J. A. (2008). Biomaterials in Orthopaedics. J. R. Soc. Interface 5 (27), 1137–1158. 10.1098/rsif.2008.0151 18667387 PMC2706047

[B87] Nguyen M. K. Jeon O. Dang P. N. Huynh C. T. Varghai D. Riazi H. (2018). RNA Interfering Molecule Delivery from *In Situ* Forming Biodegradable Hydrogels for Enhancement of Bone Formation in Rat Calvarial Bone Defects. Acta Biomater. 75 (75), 105–114. 10.1016/j.actbio.2018.06.007 29885529 PMC6119505

[B88] Nishina K. Unno T. Uno Y. Kubodera T. Kanouchi T. Mizusawa H. (2008). Efficient *In Vivo* Delivery of siRNA to the Liver by Conjugation of α-Tocopherol. Mol. Ther. 16 (4), 734–740. 10.1038/mt.2008.14 28178465

[B89] O’Brien F. J. (2011). Biomaterials & Scaffolds for Tissue Engineering. Mater. Today 14 (3), 88–95. 10.1016/S1369-7021(11)70058-X

[B90] Oryan A. Kamali A. Moshiri A. Baghaban Eslaminejad M. (2017). Role of Mesenchymal Stem Cells in Bone Regenerative Medicine: What is the Evidence?. Cells Tissues Organs 204 (2), 59–83. 10.1159/000469704 28647733

[B91] Pan Z. Ding J. (2012). Poly(lactide- co -glycolide) Porous Scaffolds for Tissue Engineering and Regenerative Medicine. Interface Focus 2 (3), 366–377. 10.1098/rsfs.2011.0123 23741612 PMC3363019

[B92] Peng S. Cao L. He S. Zhong Y. Ma H. Zhang Y. (2018). An Overview of Long Noncoding RNAs Involved in Bone Regeneration from Mesenchymal Stem Cells. Stem Cell Int. 2018, 1–11. 10.1155/2018/8273648 PMC5829309 29535782

[B93] Perez-Puyana V. Jiménez-Rosado M. Romero A. Guerrero A. (2020). Polymer-Based Scaffolds for Soft-Tissue Engineering. Polymers 12 (7), 1566. 10.3390/polym12071566 PMC7408565 32679750

[B94] Ponting C. P. Oliver P. L. Reik W. (2009). Evolution and Functions of Long Noncoding RNAs. Cell 136 (4), 629–641. 10.1016/j.cell.2009.02.006 19239885

[B95] Prakash T. P. Allerson C. R. Dande P. Vickers T. A. Sioufi N. Jarres R. (2005). Positional Effect of Chemical Modifications on Short Interference RNA Activity in Mammalian Cells. J. Med. Chem. 48 (13), 4247–4253. 10.1021/jm050044o 15974578

[B96] Qu B. Xia X. Wu H.-H. Tu C.-Q. Pan X.-M. (2014). PDGF-Regulated miRNA-138 Inhibits the Osteogenic Differentiation of Mesenchymal Stem Cells. Biochem. Biophys. Res. Commun. 448 (3), 241–247. 10.1016/j.bbrc.2014.04.091 24792185

[B97] Raftery R. M. Walsh D. P. Castaño I. M. Heise A. Duffy G. P. Cryan S.-A. (2016). Delivering Nucleic-Acid Based Nanomedicines on Biomaterial Scaffolds for Orthopedic Tissue Repair: Challenges, Progress and Future Perspectives. Adv. Mater. 28, 5447–5469. 10.1002/adma.201505088 26840618

[B98] Ramasubramanian A. Jeeawoody S. Yang F. (2015). Gene Delivery of Osteoinductive Signals to a Human Fetal Osteoblast Cell Line Induces Cell Death in a Dose-Dependent Manner. Drug Deliv. Transl. Res. 5 (2), 160–167. 10.1007/s13346-013-0163-x 25787741

[B99] Ren Z. Ma S. Jin L. Liu Z. Liu D. Zhang X. (2017). Repairing a Bone Defect with a Three-Dimensional Cellular Construct Composed of a Multi-Layered Cell Sheet on Electrospun Mesh. Biofabrication 9 (2), 025036. 10.1088/1758-5090/aa747f 28631613

[B100] Rescignano N. Fortunati E. Montesano S. Emiliani C. Kenny J. M. Martino S. (2014). PVA Bio-Nanocomposites: A New Take-Off Using Cellulose Nanocrystals and PLGA Nanoparticles. Carbohydr. Polym. 99, 47–58. 10.1016/j.carbpol.2013.08.061 24274478

[B101] Roseti L. Parisi V. Petretta M. Cavallo C. Desando G. Bartolotti I. (2017). Scaffolds for Bone Tissue Engineering: State of the Art and New Perspectives. Mater. Sci. Eng. C Mater. Biol. Appl. 78, 1246–1262. 10.1016/j.msec.2017.05.017 28575964

[B102] Ryan E. J. Ryan A. J. González-Vázquez A. Philippart A. Ciraldo F. E. Hobbs C. (2019). Collagen Scaffolds Functionalised with Copper-Eluting Bioactive Glass Reduce Infection and Enhance Osteogenesis and Angiogenesis Both *In Vitro* and *In Vivo* . Biomaterials 197, 405–416. 10.1016/j.biomaterials.2019.01.031 30708184

[B103] Sahay G. Alakhova D. Y. Kabanov A. V. (2010). Endocytosis of Nanomedicines. J. Control. Release 145 (3), 182–195. 10.1016/j.jconrel.2010.01.036 20226220 PMC2902597

[B104] Sartori E. Neves A. Magro-Filho O. Mendonça D. Krebsbach P. Cooper L. (2019). The Role of MicroRNAs in the Osseointegration Process. Int. J. Oral Maxillofac. Implants 34 (2), 397–410. 10.11607/jomi.6581 30883619

[B105] Schroeder A. Levins C. G. Cortez C. Langer R. Anderson D. G. (2010). Lipid-Based Nanotherapeutics for siRNA Delivery. J. Intern. Med. 267 (1), 9–21. 10.1111/j.1365-2796.2009.02189.x 20059641 PMC5308083

[B106] Seal B. Otero T. C. Panitch A. (2001). Polymeric Biomaterials for Tissue and Organ Regeneration. Mater. Sci. Eng. R Rep. 34 (4), 147–230. 10.1016/S0927-796X(01)00035-3

[B107] Shahabipour F. Oskuee R. K. Dehghani H. Shokrgozar M. A. Aninwene G. E. Bonakdar S. (2020). Cell-cell Interaction in a Coculture System Consisting of CRISPR /Cas9 Mediated GFP Knock‐in HUVECs and MG ‐63 Cells in Alginate‐GelMA Based Nanocomposites Hydrogel as a 3D Scaffold. J. Biomed. Mater. Res. 108 (8), 1596–1606. 10.1002/jbm.a.36928 32180319

[B108] Shen B. Zhang W. Zhang J. Zhou J. Wang J. Chen L. (2014). Efficient Genome Modification by CRISPR-Cas9 Nickase with Minimal Off-Target Effects. Nat. Methods 11 (4), 399–402. 10.1038/nmeth.2857 24584192

[B109] Shi K. Lu J. Zhao Y. Wang L. Li J. Qi B. (2013). MicroRNA-214 Suppresses Osteogenic Differentiation of C2C12 Myoblast Cells by Targeting Osterix. Bone 55 (2), 487–494. 10.1016/j.bone.2013.04.002 23579289

[B110] Shi L. Shi L. Wang L. Duan Y. Lei W. Wang Z. (2013). The Improved Biological Performance of a Novel Low Elastic Modulus Implant. PLoS One 8 (2), e55015. 10.1371/journal.pone.0055015 23437048 PMC3578840

[B111] Shi Q. Li Y. Sun J. Zhang H. Chen L. Chen B. (2012). The Osteogenesis of Bacterial Cellulose Scaffold Loaded with Bone Morphogenetic Protein-2. Biomaterials 33 (28), 6644–6649. 10.1016/j.biomaterials.2012.05.071 22727467

[B112] Soutschek J. Akinc A. Bramlage B. Charisse K. Constien R. Donoghue M. (2004). Therapeutic Silencing of an Endogenous Gene by Systemic Administration of Modified siRNAs. Nature 432 (7014), 173–178. 10.1038/nature03121 15538359

[B113] Takayama K. Suzuki A. Manaka T. Taguchi S. Hashimoto Y. Imai Y. (2009). RNA Interference for Noggin Enhances the Biological Activity of Bone Morphogenetic Proteins *In Vivo* and *In Vitro* . J. Bone Miner. Metab. 27 (4), 402–411. 10.1007/s00774-009-0054-x 19252814

[B114] Tierney C. M. Haugh M. G. Liedl J. Mulcahy F. Hayes B. O’Brien F. J. (2009). The Effects of Collagen Concentration and Crosslink Density on the Biological, Structural and Mechanical Properties of Collagen-GAG Scaffolds for Bone Tissue Engineering. J. Mech. Behav. Biomed. Mater. 2 (2), 202–209. 10.1016/j.jmbbm.2008.08.007 19627824

[B115] Trino L. D. Bronze-Uhle E. S. Bronze-Uhle A. Lisboa-Filho P. N. Mathew M. T. George A. (2018). Titanium Surface Bio-Functionalization Using Osteogenic Peptides: Surface Chemistry, Biocompatibility, Corrosion and Tribocorrosion Aspects. J. Mech. Behav. Biomed. Mater. 81, 26–38. 10.1016/j.jmbbm.2018.02.024 29477893 PMC5871601

[B116] Trompeter H.-I. Dreesen J. Hermann E. Iwaniuk K. M. Hafner M. Renwick N. (2013). MicroRNAs miR-26a, miR-26b, and miR-29b Accelerate Osteogenic Differentiation of Unrestricted Somatic Stem Cells from Human Cord Blood. BMC Genomics 14 (1), 111. 10.1186/1471-2164-14-111 23418963 PMC3637629

[B117] Truong V. A. Hsu M.-N. Kieu Nguyen N. T. Lin M.-W. Shen C.-C. Lin C.-Y. (2019). CRISPRai for Simultaneous Gene Activation and Inhibition to Promote Stem Cell Chondrogenesis and Calvarial Bone Regeneration. Nucleic Acids Res. 47 (13), e74. 10.1093/nar/gkz267 30997496 PMC6648329

[B118] Vosen S. Rieck S. Heidsieck A. Mykhaylyk O. Zimmermann K. Plank C. (2016). Improvement of Vascular Function by Magnetic Nanoparticle-Assisted Circumferential Gene Transfer into the Native Endothelium. J. Control. Release 241, 164–173. 10.1016/j.jconrel.2016.09.024 27667178

[B119] Wang M. (2003). Developing Bioactive Composite Materials for Tissue Replacement. Biomaterials 24 (13), 2133–2151. 10.1016/s0142-9612(03)00037-1 12699650

[B120] Wang M. Ge X. Zheng Y. Wang C. Zhang Y. Lin Y. (2020). Microarray Analysis Reveals that lncRNA PWRN1 ‐209 Promotes Human Bone Marrow Mesenchymal Stem Cell Osteogenic Differentiation on Microtopography Titanium Surface *In Vitro* . J. Biomed. Mater. Res. 108, 2889–2902. 10.1002/jbm.b.34620 32447825

[B121] Wang Y. Zhang S. Benoit D. S. W. (2018). Degradable Poly(ethylene Glycol) (PEG)-Based Hydrogels for Spatiotemporal Control of siRNA/nanoparticle Delivery. J. Control. Release 287, 58–66. 10.1016/j.jconrel.2018.08.002 30077736 PMC6183067

[B122] Wang Z. Wu G. Wei M. Liu Q. Zhou J. Qin T. (2016). Improving the Osteogenesis of Human Bone Marrow Mesenchymal Stem Cell Sheets by microRNA-21-Loaded Chitosan/hyaluronic Acid Nanoparticles via Reverse Transfection. Int. J. Nanomedicine 11, 2091–2105. 10.2147/IJN.S104851 27274237 PMC4876805

[B123] Wilson R. C. Doudna J. A. (2013). Molecular Mechanisms of RNA Interference. Annu. Rev. Biophys. 42 (1), 217–239. 10.1146/annurev-biophys-083012-130404 23654304 PMC5895182

[B124] Wittrup A. Lieberman J. (2015). Knocking Down Disease: A Progress Report on siRNA Therapeutics. Nat. Rev. Genet. 16 (9), 543–552. 10.1038/nrg3978 26281785 PMC4756474

[B125] Wu N. Liu B. Du H. Zhao S. Li Y. Cheng X. (2019). The Progress of CRISPR/Cas9-Mediated Gene Editing in Generating Mouse/Zebrafish Models of Human Skeletal Diseases. Comput. Struct. Biotechnol. J. 17 (1), 954–962. 10.1016/j.csbj.2019.06.006 31360334 PMC6639410

[B126] Xia C.-F. Boado R. J. Pardridge W. M. (2009). Antibody-Mediated Targeting of siRNA via the Human Insulin Receptor Using Avidin-Biotin Technology. Mol. Pharm. 6 (3), 747–751. 10.1021/mp800194y 19093871 PMC3557857

[B127] Xie Q. Wang Z. Zhou H. Yu Z. Huang Y. Sun H. (2016). The Role of miR-135-Modified Adipose-Derived Mesenchymal Stem Cells in Bone Regeneration. Biomaterials 75, 279–294. 10.1016/j.biomaterials.2015.10.042 26513420

[B128] Yang L. Cheng P. Chen C. He H.-B. Xie G.-Q. Zhou H.-D. (2012). miR-93/Sp7 Function Loop Mediates Osteoblast Mineralization. J. Bone Miner. Res. 27 (7), 1598–1606. 10.1002/jbmr.1621 22467200

[B129] Yu R. Z. Graham M. J. Post N. Riney S. Zanardi T. Hall S. (2016). Disposition and Pharmacology of a GalNAc3-Conjugated ASO Targeting Human Lipoprotein (A) in Mice. Mol. Ther. Nucleic Acids 5 (5), e317. 10.1038/mtna.2016.26 27138177 PMC5014512

[B130] Zhang J.-F. Fu W.-M. He M.-I. Wang H. Wang W.-M. Yu S.-C. (2011). MiR-637 Maintains the Balance between Adipocytes and Osteoblasts by Directly Targeting Osterix. Mol. Biol. Cell 22 (21), 3955–3961. 10.1091/mbc.e11-04-0356 21880893 PMC3204058

[B131] Zhang W. De La Vega R. E. Coenen M. J. Müller S. A. Peniche Silva C. J. Aneja M. K. (2019). An Improved, Chemically Modified RNA Encoding BMP-2 Enhances Osteogenesis *In Vitro* and *In Vivo* . Tissue Eng. Part A 25 (1–2), 131–144. 10.1089/ten.tea.2018.0112 30009674

[B132] Zhang Y. Ma W. Zhan Y. Mao C. Shao X. Xie X. (2018). Nucleic Acids and Analogs for Bone Regeneration. Bone Res. 6, 37. 10.1038/s41413-018-0042-7 30603226 PMC6306486

[B133] Zhao Y. Ding S. (2007). A High-Throughput siRNA Library Screen Identifies Osteogenic Suppressors in Human Mesenchymal Stem Cells. Proc. Natl. Acad. Sci. U.S.A. 104 (23), 9673–9678. 10.1073/pnas.0703407104 17535907 PMC1887565

[B134] Zheng Y. Zheng Y. Jia L. Zhang Y. Lin Y. (2020). Integrated Analysis of lncRNA-mRNA Networks Associated with an SLA Titanium Surface Reveals the Potential Role of HIF1A-AS1 in Bone Remodeling. RSC Adv. 10, 20972–20990. 10.1039/d0ra01242d PMC9054372 35517763

[B135] Zhu S. Zhu Y. Wang Z. Liang C. Cao N. Yan M. (2020). Bioinformatics Analysis and Identification of Circular RNAs Promoting the Osteogenic Differentiation of Human Bone Marrow Mesenchymal Stem Cells on Titanium Treated by Surface Mechanical Attrition. PeerJ 8, e9292. 10.7717/peerj.9292 32742764 PMC7365136

